# Exploring *Rhodospirillum rubrum* response to high doses of carbon monoxide under light and dark conditions

**DOI:** 10.1007/s00253-024-13079-5

**Published:** 2024-03-11

**Authors:** Manuel S. Godoy, Irene Verdú, Santiago R. de Miguel, José D. Jiménez, M. Auxiliadora Prieto

**Affiliations:** 1 Polymer Biotechnology Lab, Biological Research Centre Margarita Salas, Spanish National Research Council (CIB-CSIC), Madrid, Spain; 2 Interdisciplinary Platform for Sustainable Plastics Towards a Circular Economy-CSIC (SusPlast-CSIC), Madrid, Spain; 3 Present address: Drexel University, Philadelphia, Pennsylvania USA

**Keywords:** *Rhodospirillum rubrum*, Carbon monoxide, Syngas, Polyhydroxyalkanoates, Acetate

## Abstract

**Abstract:**

Environmental concerns about residues and the traditional disposal methods are driving the search for more environmentally conscious processes, such as pyrolysis and gasification. Their main final product is synthesis gas (syngas) composed of CO, CO_2_, H_2_, and methane. Syngas can be converted into various products using CO-tolerant microorganisms. Among them, *Rhodospirillum rubrum* is highlighted for its biotechnological potential. However, the extent to which high doses of CO affect its physiology is still opaque. For this reason, we have studied *R. rubrum* behavior under high levels of this gas (up to 2.5 bar), revealing a profound dependence on the presence or absence of light. In darkness, the key variable affected was the lag phase, where the highest levels of CO retarded growth to more than 20 days. Under light, *R. rubrum* ability to convert CO into CO_2_ and H_2_ depended on the presence of an additional carbon source, such as acetate. In those conditions where CO was completely exhausted, CO_2_ fixation was unblocked, leading to a diauxic growth. To enhance *R. rubrum* tolerance to CO in darkness, a UV-accelerated adaptive laboratory evolution (UVa-ALE) trial was conducted to isolate clones with shorter lag phases, resulting in the isolation of clones 1.4-2B and 1.7-2A. The adaptation of 1.4-2B was mainly based on mutated enzymes with a metabolic function, while 1.7-3A was mostly affected at regulatory genes, including the anti-repressor PpaA/AerR. Despite these mutations having slight effects on biomass and pigment levels, they successfully provoked a significant reduction in the lag phase (−50%).

**Keypoints:**

• *CO affects principally R. rubrum lag phase (darkness) and growth rate (light)*

• *CO is converted to CO*_*2*_*/H*_*2*_ *during acetate uptake and inhibits CO*_*2*_ *fixation (light)*

• *UVa-ALE clones showed a 50% reduction in the lag phase (darkness)*

**Graphical Abstract:**

**Figure:**
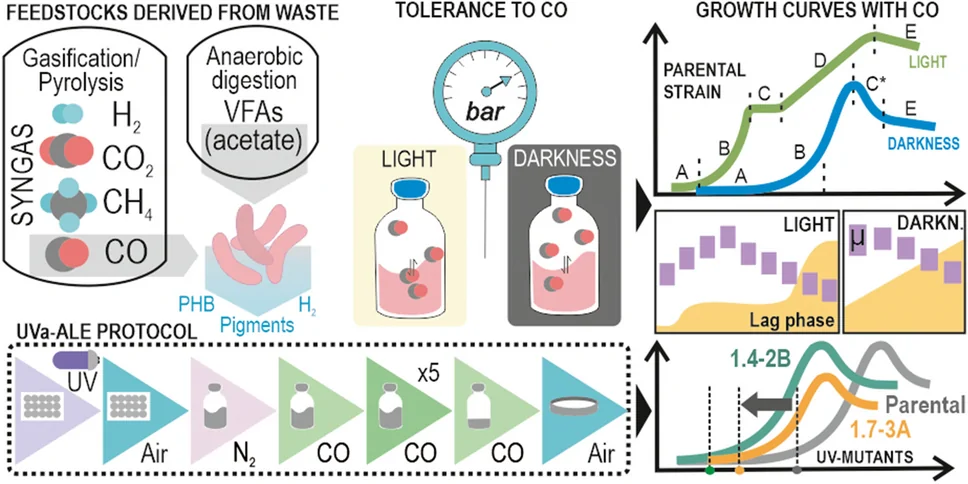

**Supplementary Information:**

The online version contains supplementary material available at 10.1007/s00253-024-13079-5.

## Introduction

Anthropogenic residues, particularly those resistant to natural degradation, are a significant concern due to their profound impact on the environment. Different strategies have been employed to address the disposal of products at its end of their life, including burial and incineration. While the former generates pollution and consumes lands rendering them unusable for productive purposes, the latter, in spite of reducing waste volume, requires a considerable amount of energy and releases detrimental gases such as CO_2_, contributing to the greenhouse effect (Makarichi et al. 2018). A combination of strategies within the framework of the circular economy paradigm can help address the challenges of end-of-life waste management. Recycling, biodegradation, gasification, and pyrolysis play a central role in this context. The latter two methods are considered environmentally responsible approaches for dealing with complex waste materials in a sustainable manner. They have different characteristics, but share the capacity to generate synthesis gas (syngas), a mixture of gases mainly composed of CO, CO_2_, H_2_, and methane (Xie et al. 2012).

Syngas serves as a valuable feedstock due to its potential to be transformed into various products, such as acetic acid, butyric acid, ethanol, and butanol, through both chemical and biological catalyses. These conversions can be achieved through chemical catalytic processes (e.g., Fischer-Tropsch synthesis), or through biological conversion methods. However, biological approaches are generally regarded as more advantageous, as they offer several benefits over chemical processes. Compared to traditional inorganic mineral-based catalysts, microbial fermentations generally offer the following benefits: (i) they can operate at lower temperatures, closer to standard environmental conditions; (ii) the process is more robust against variations in the CO/H_2_ ratio; (iii) they are less sensitive to trace amounts of contaminants in the syngas, such as char, tar, ash, chlorine, and sulfur; (iv) they tend to be more product-specific (Drzyzga et al. 2015).

In this context, the microorganisms utilized for biological conversion must meet the criteria of tolerating CO and producing industrially relevant compounds. The capability to fix CO_2_ is crucial if the objective is to contribute to carbon capture and utilization efforts. From these restrictions, tolerance to CO poses the most demanding challenge in bioprocess design since carbon monoxide is typically toxic (Davidge et al. 2009). Microorganisms able to tolerate and consume CO are referred to as carboxydotrophs. This group of microorganisms is composed of bacteria and archaea with the ability to utilize CO as a source of energy and/or carbon (King and Weber 2007). Carboxydotrophs are often found in environments such as hydrothermal vents, volcanic areas, and anaerobic habitats where CO is naturally produced. They play a significant role in carbon cycling and contribute to the overall balance of this element in various ecosystems (Brady et al. 2015).

Within carboxydotrophs, purple non-sulfur bacteria (PNSB) can metabolize CO anaerobically. A prominent member of PNSB is *Rhodospirillum rubrum*, which has served as a model organism in numerous studies investigating the synthesis of PHA. By employing the water-gas shift reaction, *R. rubrum* oxidizes CO with H_2_O to produce CO_2_ and H_2_ (Drennan et al. 2001). This process involves a carbon monoxide dehydrogenase (CODH) and allows the bacterium to obtain energy and carbon. CO_2_ can enter the Calvin cycle through ribulose 1,5-bisphosphate carboxylase (RuBisCO). However, in photoheterotrophic conditions, other carboxylases besides RuBisCO may also facilitate the conversion of inorganic carbon into central precursor molecules (Revelles et al. 2016). From a biotechnological perspective, *R. rubrum* possesses great potential. It was shown to be a promising producer of bioplastics, in particular poly(3-hydroxybutyrate-co-3-hydroxyvalerate) (PHBV) from fructose during anaerobic growth (Godoy et al. 2023a). It also has applications in the field of renewable energy, as a biocatalyst for hydrogen production (Rodríguez et al. 2021; Zhu and Li 2010). *R. rubrum* can be a source of pigments and vitamins (Wang et al. 2012) that many industries (cosmetic, food, pharmaceutical) demand.

Additionally, *R. rubrum* can consume volatile fatty acids (VFAs), a group of short-chain fatty acids consisting of six or fewer carbon atoms (acetate, propionate, butyrate, etc) which can be distilled at atmospheric pressure and are the final product of the anaerobic digestion of organic matter (Lee et al. 2014). If propionate is available, it can enter the tricarboxylic acid cycle after its conversion to succinate. This process requires the activation of propionyl-CoA and its carboxylation to methylmalonyl-CoA (Knight 1962). In the case of butyrate and acetate assimilation, the ethylmalonyl-CoA (EMC) pathway plays a key role. However, it has been observed that other metabolic routes assist the latter. For instance, a so-called methylbutanoyl-CoA pathway (MBC) has been proposed to contribute to butyrate assimilation (De Meur et al. 2020). Additionally, pyruvate:ferredoxine oxidoreductase (PFOR) overexpression in the presence of acetate could aid its assimilation via the reductive carboxylation of acetyl-CoA into pyruvate (Leroy et al. 2015). Polyhydroxyalkanoates synthesis is crucial for maintaining the intracellular redox state, especially with high light intensity when acetate is the carbon source (Bayon-Vicente et al. 2020).

On the other hand, If the source of carbon and/or energy is supplied as a gas, as is the case of cultures grown from syngas, the partial pressure is the key parameter to control the solubility, and therefore its bioavailability. In terms of growth effects, CO substrate toxicity has not been observed previously in this species (Karmann et al. 2019); however, a proteomic study showed that CO could affect cofactor biosynthesis, especially metallic cofactors (Cavazza et al. 2022). To the best of our knowledge, the highest partial pressure of CO to which *R. rubrum* has been exposed was around 1 bar (Rodríguez et al. 2021). Growth in higher doses of this gas has not been reported so far. The relevance of this knowledge lies in the necessity of maximizing the gas rate transfer to the liquid to speed up growth and bioproduct formation.

To address this question, in this work, the behavior of *R. rubrum* grown in different partial pressures of CO has been explored under light and darkness conditions. The VFA acetate was chosen as a co-substrate to promote PHA accumulation (Narancic et al. 2016). We analyzed culture evolution in parameters such as biomass and PHB production, acetate and CO consumption, and H_2_/CO_2_ generation in selected levels of this gas to have a detailed characterization in defined metabolic scenarios. We also designed and performed a successful UVa-ALE to obtain strains that could overcome faster the tough adaptation barrier imposed by high doses of CO in darkness, obtaining two clones that fulfilled this objective. This work represents a solid contribution to the understanding of *R. rubrum* physiology under a rich CO atmosphere both under light and in darkness, and provides valuable data for biotechnological approaches based on syngas exploitation.

## Materials and methods

### Strains and growth conditions

**Fig. 1:**
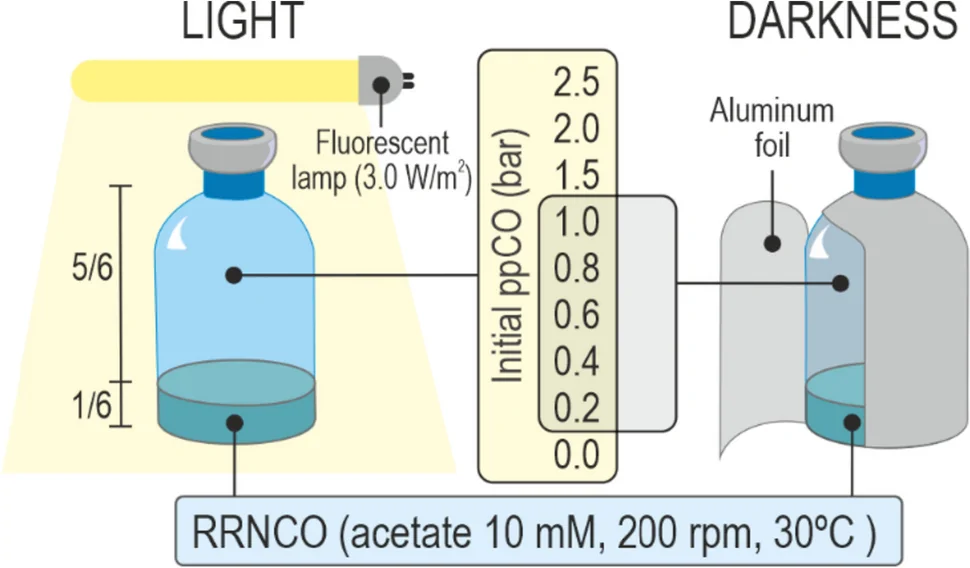
Scheme of the general experimental design used in this study. Cultures were grown in RRNCO medium (acetate 10 mM) and different amounts of CO. For cultures grown under light, the gas was added in a range from 0.0 to 2.5 bar. In the case of cultures grown in darkness, this range was from 0.2 to 1.0 bar, since in the absence of light, *R. rubrum* shows no growth without CO when acetate is the main carbon source and the lag phase was excessively long (> 25 days) for levels of CO higher than 1.0 bar. To avoid the accidental interference of light in dark conditions, bottles were covered with aluminum foil

The strain used in this work was R02_01, a derivative of the wild-type strain ATCC 11170 (DSMZ ref. no. 467T, here referred to as strain S1). Rr02_01 was isolated and characterized previously in our laboratory due to its impaired pigment production under low oxygen levels (Godoy et al. 2023b). Strains were stored at −80°C in Luria-Bertani (LB) medium supplemented with 15% v/v glycerol. Cultures were performed using RRNCO (*R. rubrum* no-light carbon monoxide) medium (Kerby et al. 1995) containing acetate 10 mM and no biotin, as no big difference was observed when omitting this vitamin (data not shown). Experimental cultures were conducted in serum bottles (60 or 120 mL) occupying 1/6 of the total flask volume with medium (10 or 20 mL respectively) (Fig. 1). Before autoclaving, bottles were heated (70–80°C approx.) in a microwave oven and subsequently degassed by flashing with N_2_ for 15–20 min. After this, bottles were sealed with 20-mm-thick chlorobutyl plugs (Wheaton® W224100-202) and secured with metal clamps (Wheaton® 12433738). Following sterilization, once at room temperature, a syringe was used to add phosphate (19.1 mM), acetate (10 mM), and the reducing agent sodium sulfite (0.01%). Once the culture bottles were prepared as detailed previously, the different gases were added. The N_2_ present in the bottles (from the bubbling of the culture medium prior to autoclaving) was extracted using a vacuum pump (Fisher Scientific Vacuum Pump), under a fume hood. After this, a mixture of N_2_:Ne (1:1), representing 1/5 of the total volume of the headspace, and variable volumes of CO were added in the gas phase. It must be noted that when NH_4_^+^ is supplied in excess (as is the case in the RRNCO medium), the nitrogen fixation machinery is not induced by the presence of N_2_ (Schultz et al. 1985). Thus, N_2_ is not expected to be used as a nitrogen source under these conditions. The total pressure of the bottles was levelled with He to 1.2 bar (darkness) or 2.5 bar (light). In the case of cultures grown in darkness, the condition without CO was omitted, as *R. rubrum* requires this gas as an energy source when acetate is the carbon source and no light is provided. Higher levels of CO were also omitted, as the lag phase became excessively long (> 25 days) for levels of CO higher than 1.0 bar. The internal pressure of each bottle was tested using a digital manometer (Mano LEO 1, Keller ®).

The pre-cultures were done under light, with acetate and N_2_. Before inoculation of the cultures, cells from the pre-culture were centrifuged (16.000 rcf, 5 min), washed, and resuspended with saline solution (0.8% w/v NaCl). Experiments were initiated with an OD_660_ of 0.05 and incubated at 30°C, 200 rpm, either under light or in darkness. Cultures that required dark conditions were incubated in bottles covered with aluminum foil to avoid the inadvertent incidence of light. Phototrophic cultures were exposed to luminescent lamps (Philips Master TL-D 18W/865, 6500 K), and the light intensity (2300 lux or ~31 μmol·m^−2^·s^−1^) was measured with a Lutron LX-102 Electronic Light Meter (a brief comparison of light usage in various studies is available in Table S1).

### Characterization of bacterial growth, biomass determination, and pigment production

Cell growth was assessed by measuring the turbidity (OD_660_) of the cultures using a MultiskanSky spectrophotometer (Thermo Fisher Scientific®) with a 96-well plate. The OD_660_ values presented in this study were adjusted by considering the optical path length. The levels of the complex antenna, referred to as photo-membrane production (PMP), were determined by normalizing the optical density at 880 nm to the OD_660_ (Abs_880/660_) (Grammel and Ghosh 2008), using the same instrument. The growth rate (*μ*) was determined as the slope of linear regression of the natural logarithmic values of the relation OD_x_/OD_initial_ vs. time during the growth phase. The lag phase was estimated as the time point where this line intercepts the horizontal axis (Dalgaard and Koutsoumanis 2001). Final biomass was determined gravimetrically by weighing the lyophilized pellet of the cultures. Total protein content was assessed using the Bradford method (Bradford 1976). For this purpose, 100 μL of 0.1 M NaOH was added to an equal volume of the culture sample and heated at 94°C for 10 min in closed Eppendorf tubes. Subsequently, 20 μL of the properly diluted mixture was added to 200 μL of the reagent in a 96-well plate. Measurements were conducted using a MultiskanSky spectrophotometer (595 nm). A standard curve was generated using known solutions of bovine serum albumin (Merk, A9418). The values of residual biomass (without PHB) were used to establish a correlation curve between the weight of dried pellets taken at the stationary phase (with no PHB) and the protein content of the sample. These values were used to estimate the CDW for the % PHB.

### Quantification of polyhydroxyalkanoates

Cellular PHA content was determined by the crotonic acid method where the polyester content is quantified by the hydrolyzation and transformation of the constitutive monomers (3-hydroxybutyrate) into crotonic acid (Watanabe et al. 2012). Briefly, the biomass of 800 μL of culture was resuspended in a solution of NaOH (1M). This mixture was incubated at 94°C for an hour and subsequently neutralized by introducing an equivalent volume of HCl (1N). The neutralized samples were centrifuged at 2600 rcf for 10 min. The resulting supernatants were filtered through 0.45 μm pore filters (13 mm, Avantor Ref. No. 514-0069). The identification and quantification of crotonic acid were carried out through high-performance liquid chromatography (HPLC) employing the Agilent® 1260 Infinity II LC System. A calibrated standard curve using commercial PHB (Sigma Aldrich®) dissolved in chloroform (0.5 g·L^−1^) was employed to estimate the concentrations of the experimental samples. The crotonic method was validated using methanolysis and gas chromatography coupled with mass spectrometry (GC-MS) (Godoy et al. 2023a).

### High-performance liquid chromatography determinations

The culture samples underwent centrifugation, and the supernatant was filtered following the same procedure used for PHA hydrolysates (as described above). They were analyzed using high-performance liquid chromatography (HPLC) with an Agilent® 1260 Infinity II LC System and an Aminex HPX-87H column from Biorad, maintained at 50°C. The mobile phase employed was H_2_SO_4_ (5 mM), with a flow rate of 0.6 ml·min^−1^. Detection was carried out using a refractive index detector and a UV detector at 210 nm. Compounds were identified based on their retention time, and their concentration was estimated using standard curves prepared with HPLC-grade purity compounds (Merk®). For PHA quantification (crotonic acid), the same chromatographic conditions were applied, with the exception that the column was heated to 60°C. In this particular case, peak detection was solely performed using the UV detector.

### Gas determination

The composition of the gas phase of the cultures was characterized by gas chromatography (GC), using the gas chromatograph (Agilent 7890A GC), equipped with a thermal conductivity conductor (TCD) and two columns connected in series (80/100 Porapak Q and 70/80 Molesieve 13X). The procedure is described elsewhere (Revelles et al. 2016). Samples were collected in 10 ml HS vials (Agilent®) previously purged with He. The gas analyzer was calibrated with the same mobile phase (He), and a standard line was prepared from samples with known amounts of syngas, He and Ne. Quantitative and qualitative gas characterization was carried out using GC data analysis software (ChemStation rev. B.04.03-SP1; Agilent Technologies, Icn.).

### Mutagenesis protocol

All the cultures which took part in the mutagenesis protocol were conducted in the absence of light. Cells of *R. rubrum* Rr02_01 grown to the mid-exponential phase (OD_660_ ~ 1.0) in Van Niel’s medium (K_2_HPO_4_ 1 g·L^−1^, MgSO_4_ 0.5 g·L^−1^, yeast extract 10 g·L^−1^, pH 7.0–7.2) (Revelles et al. 2016) were exposed to UV radiation (ChemiDoc XRS+, Bio-Rad, 302 nm) during 20 min using a 24-well polystyrene plate (Corning ®) loaded with 1 mL of culture. Subsequently, the cells were allowed to recover aerobically in the same rich medium without agitation for 10 h, and then with agitation (200 rpm) for 4 days. To make the transition to anaerobic growth, cells from each well were centrifuged separately and resuspended in 100 μL of saline solution. This volume was used to inoculate anaerobic bottles saturated with N_2_ containing RRNCO (acetate 10 mM). After 24 h, CO (partial pressure of CO, or ppCO 0.8 bar) was added to the bottles (first step of selective pressure) and incubated at 30°C until they were visibly turbid (OD_660_ 0.5–1.0). A fraction of this culture (1 mL) was transferred to a new bottle containing a ppCO 2.0 bar (second step of selective pressure) and incubated until they were visibly turbid (40–50 days), after which they were passed five times to new bottles with the same conditions (ppCO 2.0 bar). At the fifth passage, 1 mL of each bottle was serially diluted and transferred to bottles containing 10 mL of solid (1.5 g·L^−1^ agar) RRNCO and CO:N_2_ (4:1, at 1 bar) to maintain selective pressure. Some days after inoculation (10–20 days), colonies appeared on the surface of the solid medium. Several colonies were transferred to Van Niel’s solid medium (1.5 g·L^−1^ agar) plates in aerobic conditions, most of which did not survive. Finally, a collection of 20 clones were grown in rich medium and stored in Van Niel’s medium with glycerol (15%) at −80°C for posterior characterization.

### Genomic sequencing and genetic analysis of mutations

Genomic DNA of strains 1.4-2B and 1.7-3A was extracted using the phenol-chloroform extraction method (Ibero et al. 2019; Green and Sambrook 2017). DNA samples were sent to MicrobesNG (Birmingham, UK) for genomic sequencing. The resulting reads, including those from the genome of strain Rr02_01 previously sequenced (Godoy et al. 2023b), were aligned and compared with the reference genome of the ATCC 11170 using Geneious Prime software. Discrepancies with the reference genome were filtered by the coverage value (CV>15 reads) and the frequency of variation (FV>70%), and corroborated by Sanger sequencing (Secugen®, Madrid) using primers listed in Table S2. The sequences of the strains Rr02_01, 1.4-2B, and 1.7-3A are deposited in the SRA bank of the NCBI under the BioProject accession number PRJNA1041326.

### Statistical analysis

Data analysis was performed using MS Excel and OriginPro. Reported values are indicated as averages ± standard deviation. Normality was tested with the Shapiro-Wilk test. In the case of multiple comparisons, the level of statistical significance was evaluated by ANOVA with Dunnett’s or Tukey’s post hoc test, or by unpaired two-tailed Student’s test in the case of pairwise comparisons, as specified in the legend of the corresponding figure.

## Results

### *R. rubrum* characterization under different levels of CO in light and dark conditions

CO, which is one of the main components of syngas, has been extensively studied in *R. rubrum* (Karmann et al. 2019; Kerby et al. 1995; Rodríguez et al. 2021; Shelver et al. 1995). However, the physiological effects of different doses of CO have not been assessed in a systematic study, specially at high levels of this gas. To obtain this information, cells of *R. rubrum* Rr02_01 were cultivated in sealed bottles with different concentrations of CO in the headspace. This strain can reach higher levels of PMP than the wild-type strain S1, both under light and in darkness (Fig. S1).

### Growth kinetics

**Fig. 2:**
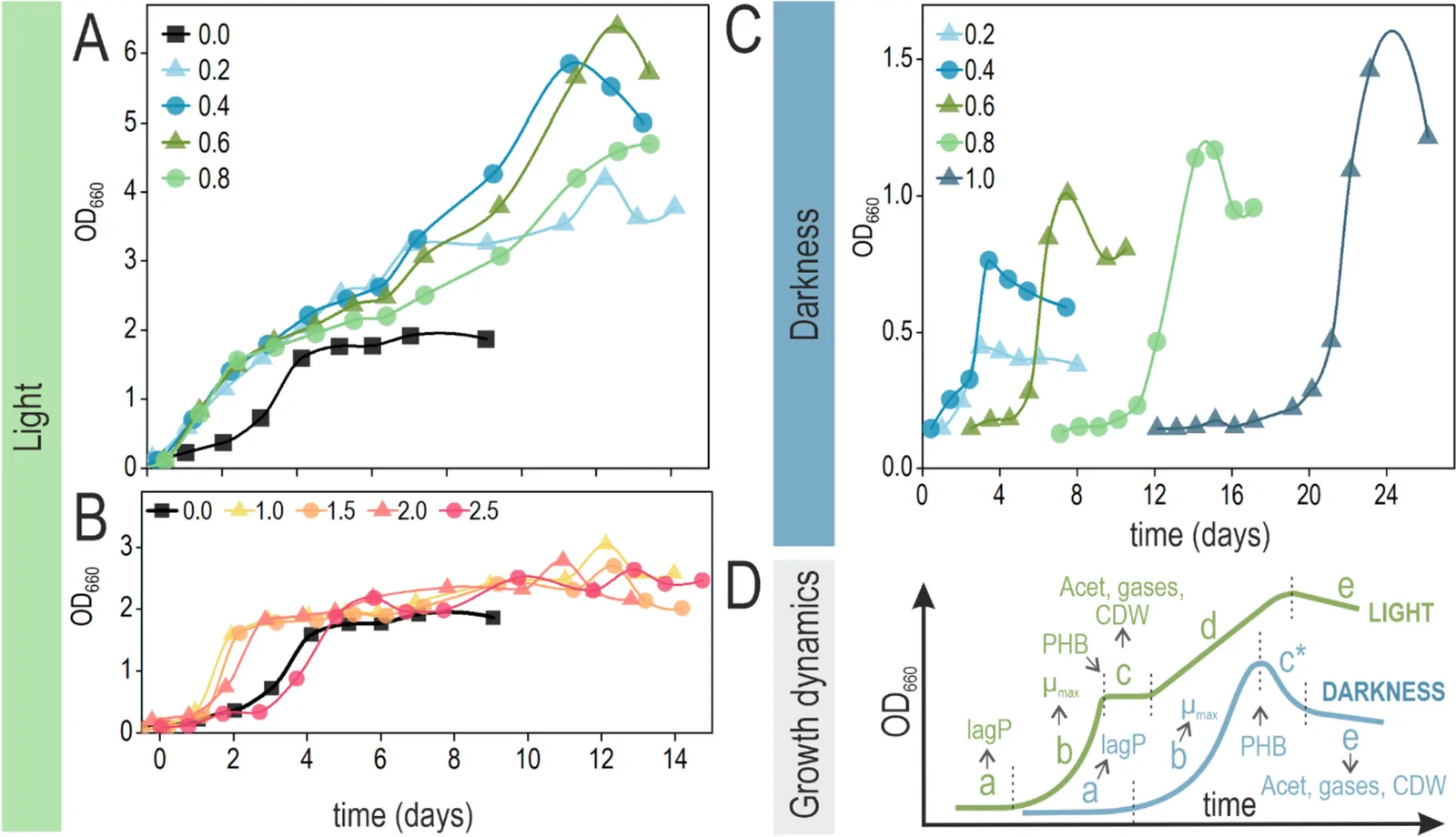
Growth curves under different levels of CO with and without light. Phototrophic cultures grown with an initial ppCO ranging from (A) 0.0 to 0.8 bar and from (B) 1.0 to 2.5 bar. The condition of ppCO = 0.0 bar is displayed in both graphs to facilitate the comparison. (C) Cultures grown in darkness with an initial ppCO ranging from 0.2 to 1.0 bar. Curves represent the mean value of OD_660_ (*n* = 3). Standard deviation was less than 10% in all cases. (D) A schematic representation of the growth dynamics observed in this experiment is depicted: (*a*) Initial lag phase; (*b*) exponential growth (μmax); (*c*) turbidity remains steady (light) or c*) rapidly decreases (darkness). (*d*) Some cultures (light, ppCO 0.2–0.8 bar) exhibit a second growth phase with a *μ* < *μ*_max_ after phase C. (*e*) Turbidity decreases gradually (stationary phase). In the case of phototrophic cultures with ppCO = 0.0 bar or ≥ 1.0 bar, there is no *d* transition between *c* and *e*. The timepoints/lapses where the different parameters were measured are indicated with grey arrows. LagP, lag phase; Acet, acetate

Through the range of initial ppCO explored in photoheterotrophic conditions, two different behaviors in the growth kinetics can be noticed (Fig. 2). The simplest one could be associated with the canonical bacterial growth curve, with an initial lag phase, followed by exponential growth and concluded with a stationary phase. Cultures exposed to light with an initial ppCO of 0.0 bar and 1.0 to 2.5 bar fit in this type of curve. However, an extended growth limit was observed for the range of ppCO from 0.2 to 0.8 bar. In these cases, a brief re-adaptation phase followed the exponential growth, after which cells re-started biomass formation at a slower rate, eventually reaching the stationary phase. This curve matches with a typical diauxic growth. With simpler kinetics, cultures in darkness behaved similarly despite varying initial ppCO concentrations. As acetate cannot sustain growth per se in darkness (Kerby et al. 1995), bottles without CO were not included in the experimental design. Without light, just one growth phase was observed, after which OD_600_ decreased to eventually remain steady in the stationary phase (Fig. 2C). This decrease after the maximum of turbidity could reflect lysis or, more likely, changes in their morphology/compositions triggered by the exhaustion of a limiting nutrient.

### Lag phase and growth rate

**Fig. 3:**
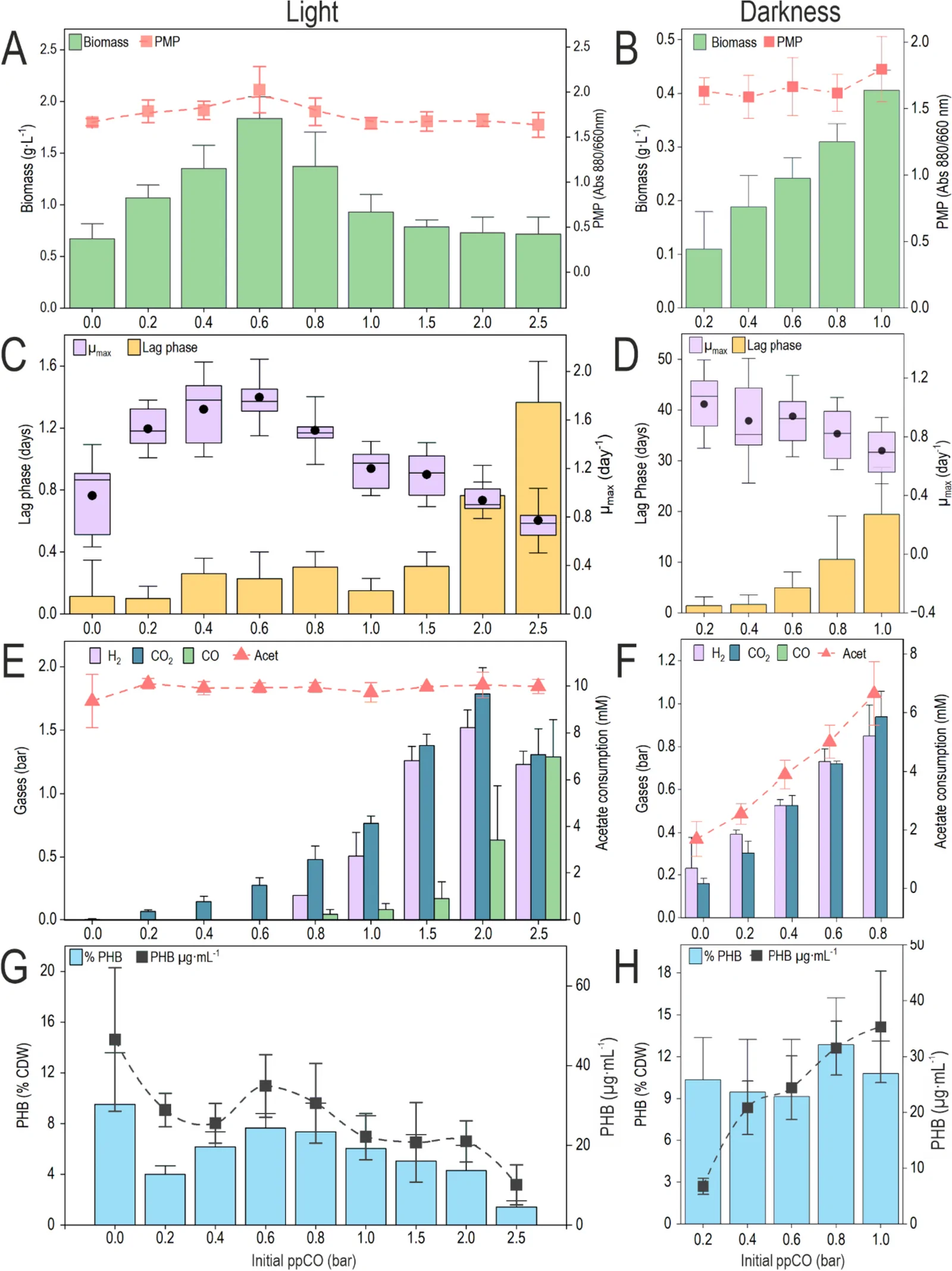
General profiling of *R. rubrum* grown under different levels of CO with and without light. Using RRNCO medium, strain Rr02_01 was cultured under increasing levels of initial ppCO, from 0.0 to 2.5 bar under light (A, C, E, G) and 0.2 to 1.0 bar in darkness (B, D, F, H). Cultures were monitored and assessed for several relevant parameters, such as the production of biomass and PMP (A and B); the growth rate and lag phase (C and D); the presence of CO_2_, H_2_, and CO in the headspace; and the consumption of acetate (E and F) and the accumulation of PHB (G and H). For more details on the sampling times, see Fig. 2C. Values represent the mean, and error bars represent the standard deviation of at least three independent biological replicates

The most conspicuous effect of CO on cell growth was related to the lag phase. In phototrophic cultures, while the lag phase remained steady for doses up to 1.5 bar, this variable increased for higher initial ppCO values, ultimately reaching 1.4 ± 0.3 days at ppCO of 2.5 bar (Fig. 3C, Fig. S2). In the case of the maximal growth rate (*μ*_max_), the optimum was obtained at ppCO 0.6 bar (1.8 ± 0.2 day^−1^), and the lowest value was for the highest level of initial ppCO (0.8 ± 0.2 day^−1^). In darkness, this effect was more pronounced (Fig. 3D). The lag phases in these conditions ranged from 1.4 ± 1.2 days (ppCO 0.2 bar) to 19.5 ± 6.1 days (ppCO 1.0 bar). The *μ*_max_ decreased in darkness in a much smoother fashion than the increase in the lag phase: from 1.1 ± 0.2 day^−1^ to 0.7 ± 0.2 day^−1^ (ppCO of 0.2 and 1.0 bar respectively). These facts reflect that the hardest challenge for cells at high doses of this gas is to adapt to CO, which once accomplished, leads to a similar evolution of the culture.

### Acetate consumption and transformation of CO into CO_2_/H_2_

The acetate (10 mM) of the cultures grown photoheterotrophically was completely depleted by the end of the first exponential growth phase (Fig. 3E). Gas consumption/production profiles varied depending on the initial ppCO. Without CO, *R. rubrum* did not produce significant amounts of any of the studied gases. In cultures fed with CO, different amounts of H_2_ and CO_2_ were produced in a CO-dependent manner: the higher the initial ppCO was, the more H_2_ and CO_2_ were accumulated in the headspace of the bottles. At the end of this experiment, cultures with an initial ppCO from 0.2 to 0.8 had no H_2_ or CO remnant. On the contrary, for higher initial ppCO levels, various amounts of H_2_, CO_2_, and CO were detected. This indicates that the consumption of H_2_ and CO_2_ occurred only in cases where CO was completely depleted from the headspace (ppCO 0.2 to 0.8 bar). H_2_ consumption to drive photosynthesis as a source of reductive power will be elaborated in the “Discussion” section.

In darkness, the final biomass and acetate consumption were also directly dependent on the initial ppCO (Fig. 3F). In this case, unlike photoheterotrophic cultures, acetate was not depleted for any initial ppCO, meaning it was in excess in respect to CO, the limiting growth factor, whereas CO was totally consumed by the end of the experiment.

### PHB accumulation and pigment production

At the end of the exponential growth in light or during the stationary phase both in light and dark cultures, the intracellular storage of PHB was almost completely depleted (data not shown). To compare the different conditions, its concentration was measured at the end of the exponential phase, when the OD_660_ was maximal. Under light, the amount of PHB was higher at ppCO 0.6 (8.3 ± 3.4 % CDW, 34.9 ± 7.9 μg·mL^−1^) compared to the other conditions where CO was present (Fig. 3G). In the absence of CO, the accumulation of PHB was similar, reaching 9.5 ± 2.7 % CDW or 46±18 μg·mL^−1^. In darkness, the upward trend on biomass along the increasing initial ppCO was followed by an upward volumetric accumulation of PHB, keeping the % PHB unvaried along the range studied (Fig. 3H). While the latter reached around 10% in the different conditions, the volumetric production of the polymer reached as much as 35 ± 13 μg·mL^−1^ with ppCO of 1.0 bar.

Observing the values of PMP represented in Fig. 3A, at the end of the first growth phase, the highest PMP was observed at ppCO of 0.6 bar (2.1 ± 0.3 Abs_880/660_) and the minimum at ppCO of 2.5 bar (1.6 ± 0.1 Abs_880/660_). This value was similar to the PMP produced in the absence of CO (1.67 ± 0.04 Abs_880/660_). Cultures grown in darkness showed a PMP similar for all the initial ppCO studied (~1.7 Abs_880/660_) (Fig. 3B).

### Kinetic analysis of main culture parameters at selected CO levels

**Fig. 4:**
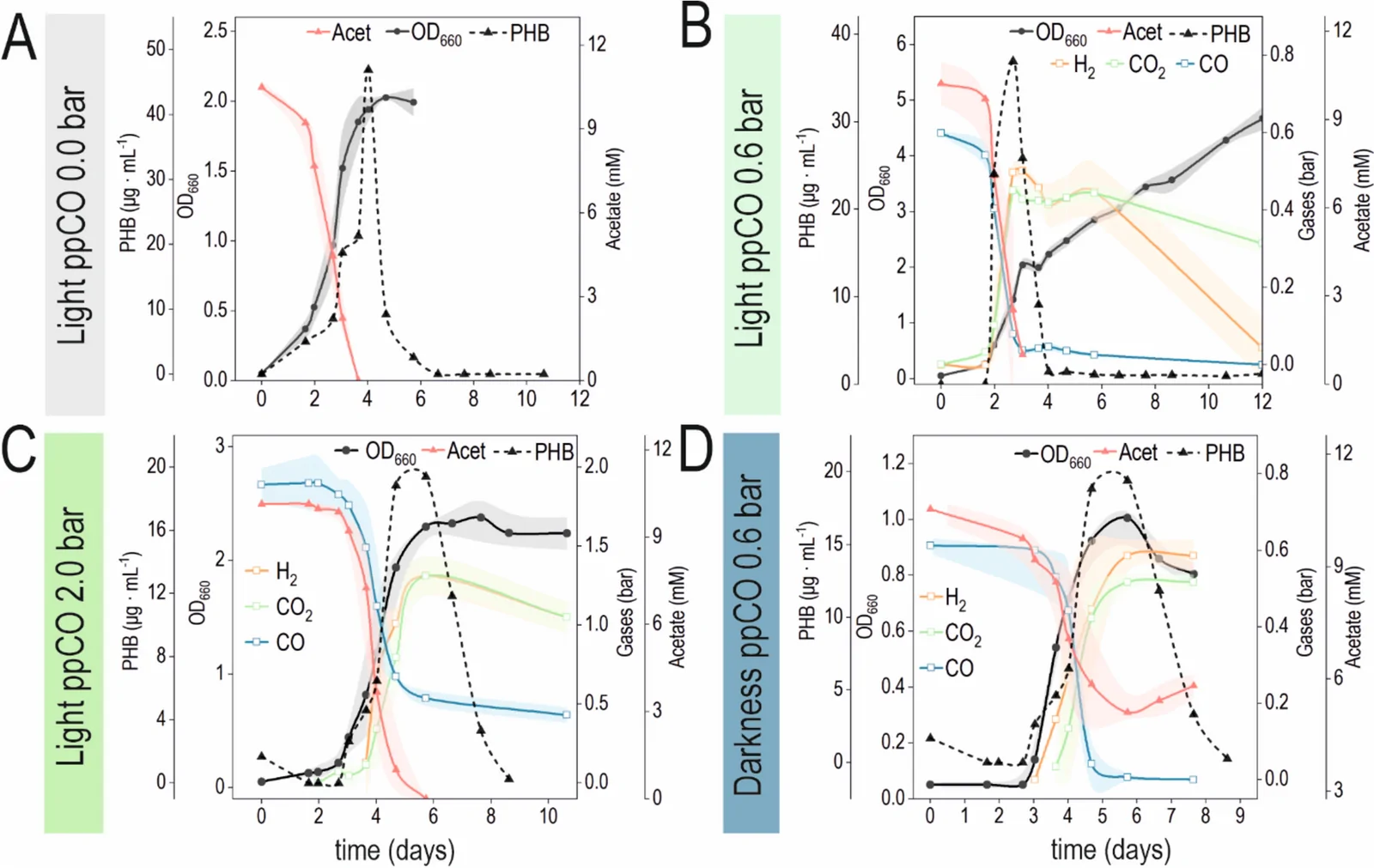
Culture evolution in selected conditions. Curves of acetate and CO consumption, OD_660_, and H_2_/CO_2_ production for each of the selected conditions. Photoheterotrophic cultures were conducted with an initial ppCO of (A) 0.0 bar, (B) 0.6 bar, or (C) 2.0 bar. No gas was detected in the condition with no CO (A). (D) Cultures in darkness were evaluated with ppCO of 0.6 bar. Curves represent the mean of at least three independent biological replicates, and the shadow denotes the standard deviation. For PHB values. The standard deviation was lower than 15% of the mean

In the previous section, two types of growth kinetics were observed for *R. rubrum* cultured under light depending on the initial ppCO. On the other hand, in darkness, no such differences were observed: all the cultures exhibited equivalent properties regarding biomass, pigment and PHB production, and the carbon substrate (acetate and CO) consumption. Consequently, we decided to zoom in on the physiological aspects of three typical growth kinetics for phototrophic cultures (ppCO of 0.0 bar, 0.6 bar, and 2.0 bar) and one grown in darkness (ppCO=0.6 bar) (Fig. 4).

As expected, acetate and CO consumption were associated with light and dark growth. For a ppCO of 0.6 bar in light, CO and acetate were depleted approximately at the same time (Fig. 4B). On the other hand, CO could not be completely consumed at the expense of acetate in cultures with an initial ppCO of 2.0 bar (Fig. 4C). Consequently, a fraction of this gas remained in the bottle until the end of the experiment. Therefore, CO depletion seems to trigger the second growth phase. When cultures run out of acetate in light, PHB undergoes a rapid mobilization. In darkness, acetate is consumed all along cell growth, when biomass and PHB are produced (Fig. 4D). When CO is over, acetate consumption ceases immediately, and its concentration increases in the supernatant, meaning this acid is secreted probably at the expense of PHB depolymerization.

To verify if the general behavior of strain Rr02_01 was similar for its parental strain S1, the growth curves of both were tested in these conditions, giving very similar results in terms of biomass formation (OD_660_), with a higher level of PMP for the strain Rr02_01 in all the conditions, as expected (Fig. S3). Under light without CO, differences were minimal. Interestingly, in cultures grown with light and ppCO 0.6 bar, strain Rr02_01 grew faster during the second growth phase than the parental strain (0.066 ± 0.009 day^−1^ vs 0.045 ± 0.004 day^−1^, respectively) and reached a higher OD (4.94 ± 0.44 vs. 3.97 ± 0.21, respectively) (Table S3). At this point, the differences with respect to the PMP were also maximal, with a PMP value 35% higher than the parental strain. Moreover, the stationary phase of S1 presented a much faster decline in the OD as can be seen in Fig. S3. These showed that while both strains behave similarly for variables such as OD and growth rate during growth in CO, after the depletion of this gas, strain Rr02_01 seems to grow more efficiently using CO_2_ and H_2_.

### Expediting adaptation to CO in darkness

Great metabolic versatility is one of the main characteristics of PNSB, and this fact is manifested in the high tolerance we observe in *R. rubrum* under CO. However, this gas has a significant impact on the physiology of *R. rubrum* during dark growth, particularly during the lag phase. To test whether we could further enhance the metabolic tolerance of *R. rubrum* in these conditions, we intended to reduce its long lag phase by randomly modifying its genetics. As a proof of concept, we decided to conduct an experiment based on adaptive laboratory evolution (ALE) principles. Given that a traditional ALE requires several generations to find positive mutations and *R. rubrum* has a high duplication time in darkness, a random mutagenesis factor was incorporated into the conventional ALE experiment to accelerate the emergence of mutant clones under high CO levels. We called this approach a UV-accelerated (UVa) ALE or simply UVa-ALE.

**Fig. 5:**
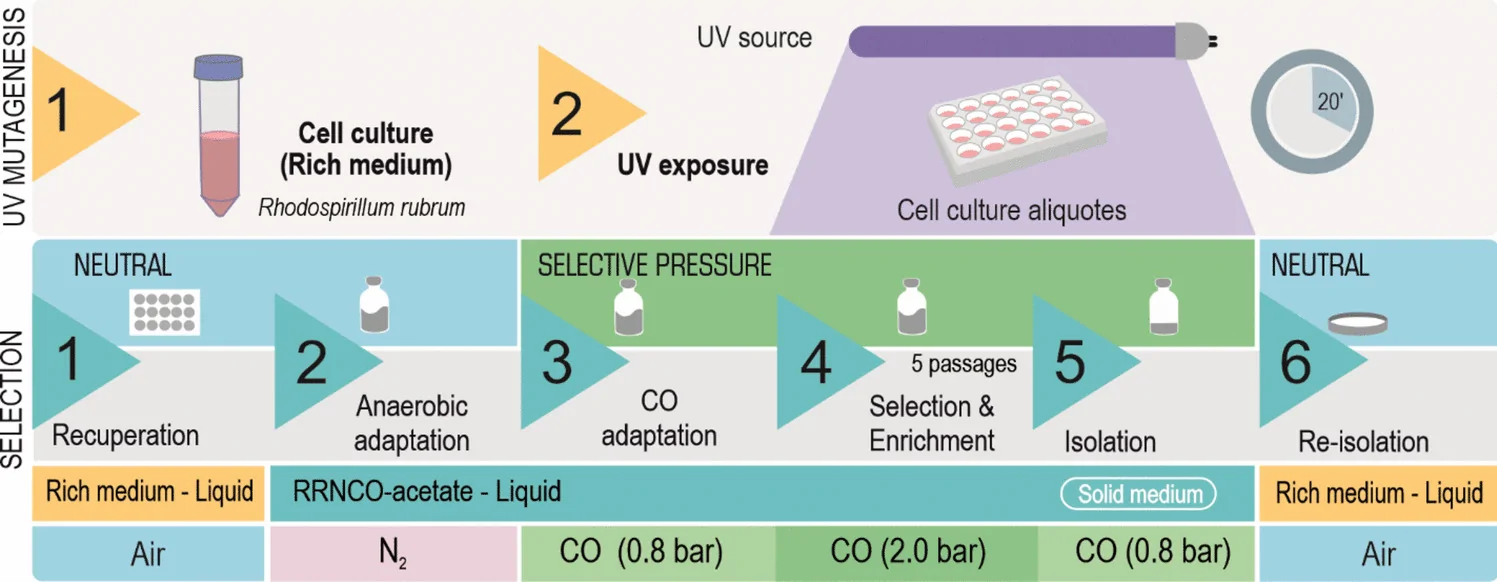
Scheme of the UVa-ALE. A culture of *R. rubrum* Rr02_01 grown in rich medium was exposed to UV for 20 min, after which, they were allowed to recuperate. The idea to use UV radiation was to augment the mutation rate, given that the replication of *R. rubrum* in CO is slow under dark conditions, making it necessary to increase the genetic variability and thus reduce the duration of the experiment. The selective pressure consisted on a first step of ppCO of 0.8, followed by other of ppCO 2.0 bar (5 passages). The isolation of clones was done in a controlled atmosphere (ppCO 0.8 bar) using solid medium

**Fig. 6:**
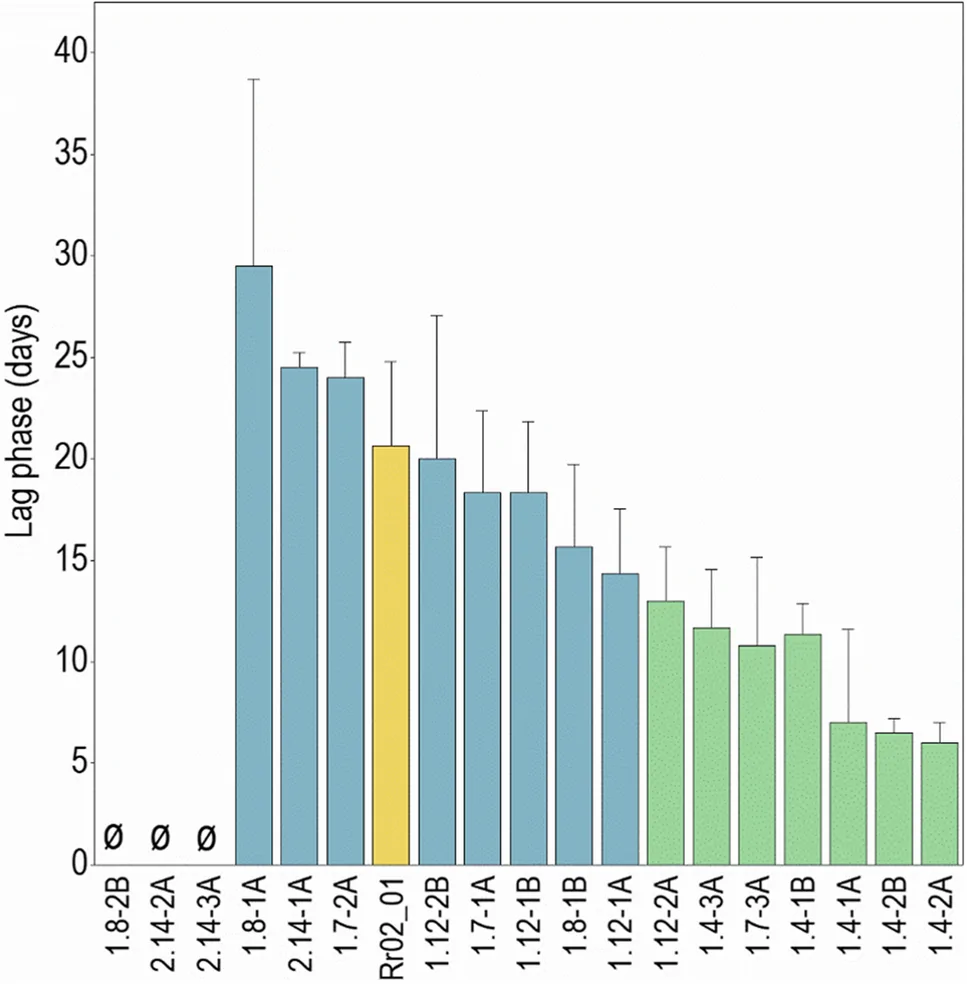
Screening of clones with shorter lag phase. 18 clones isolated after the UVa-ALE (blue) were tested and compared to the parental strain Rr02_01 (yellow) in RRNCO medium (ppCO 1.0 bar). The three clones which did not grow (ø) were discarded. Clones with a significant difference with respect to parental were colored in green (ANOVA, Dunnett’s post hoc test). The values represent the mean, and the error bars represent the standard deviation of three experimental units

A scheme of the UVa-ALE is shown in Fig. 5 (the details can be found in the “Materials and methods” section). After exposing bacteria to UV, they were allowed to recuperate aerobically in a rich medium prior to being incubated anaerobically with N_2_. This step is supposed to minimize stress before the first exposure to CO. The selective pressure was exerted in two levels (ppCO of 0.8 and 2.0 bar) to avoid an excessive stringent condition that could imply a high mortality of cells. It is worth mentioning that we had never seen the growth of *R. rubrum* in 2.0 bar of CO in our experiments when light was not supplied. The isolation of clones was done in an atmosphere of CO (0.8 bar) to maintain the selective pressure, using bottles with a solid medium (Fig. S4). In this step, 18 clones were screened in liquid RRNCO medium at an initial ppCO of 1.0 bar, from which 3 did not grow and only 7 showed a significantly shorter lag phase compared to the parental strain Rr02_01 (Fig. 6). When grown in a rich medium for routine handling, clone 1.4-2B showed more robust growth compared to clones isolated from the same bottle (series 1.4). Clone 1.7-3A was particularly interesting because it formed pigmented colonies on the agar plate, in contrast to the Rr02_01 strain (data not shown). For these reasons, clones 1.4-2B and 1.7-3A were selected for further analysis.

### Increased CO adaptability impacted PHA or pigment production

**Fig. 7:**
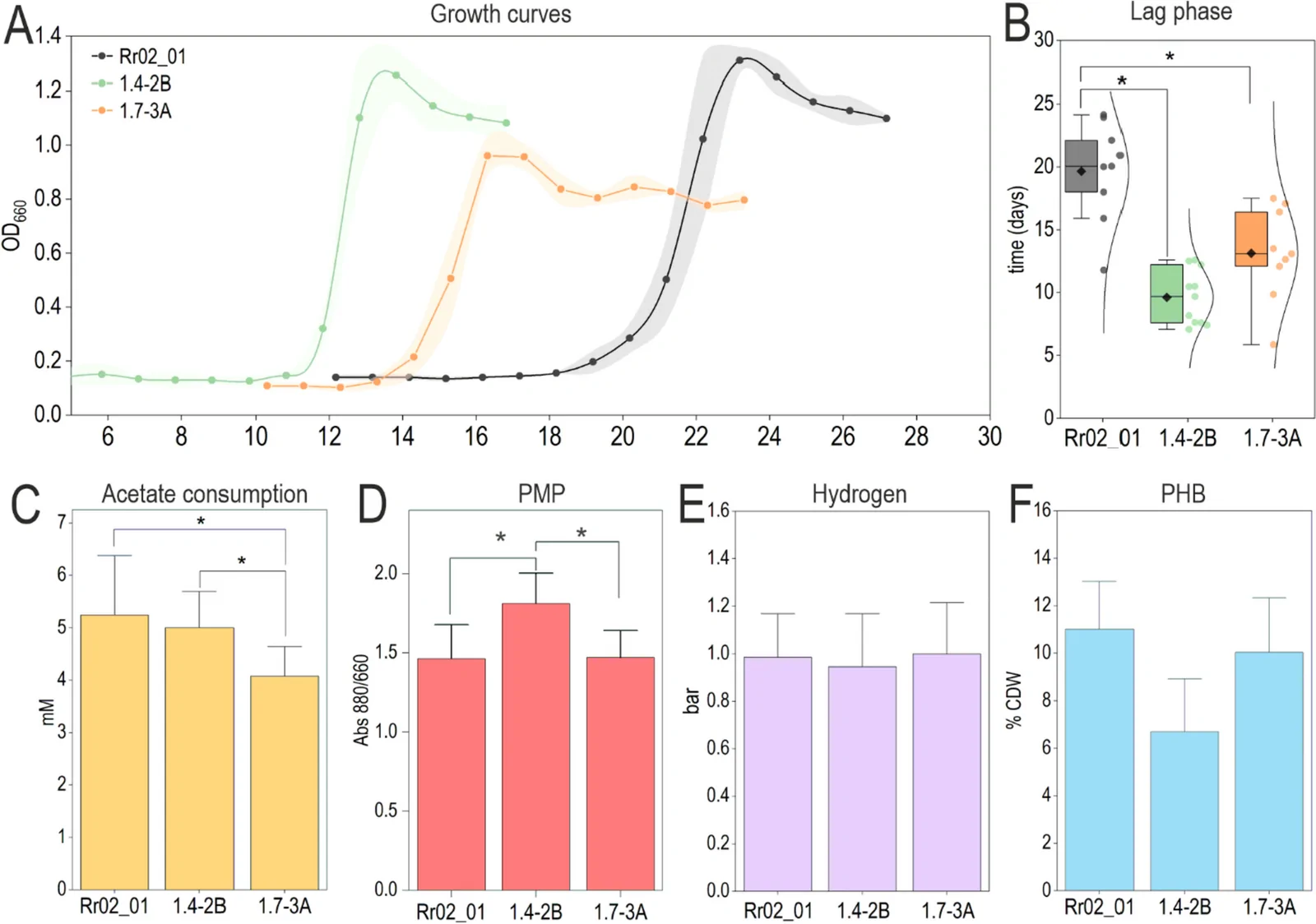
Characterization of strains 1.4-2B and 1.7-3A. (A) The growth curves are represented at the corresponding average lag phase of each strain. (B) The values of lag phase are represented with boxplots and a normal curve is fitted to the experimental data (dots). The consumption of (C) acetate and the production of (D) PMP, (E) H_2_, and (F) PHB was measured. The cultures were done in RRNCO medium with ppCO of 1.0 bar. Values represent the mean, and the error bars the standard deviation of at least three independent biological replicates. Asterisks denote significance with *p* value < 0.05 (ANOVA, Tukey’s test)

The mutations in both 1.4-2B and 1.7-3A strains might have pleiotropic effects beyond the increased adaptability to CO. Four main products were assessed to test the feasibility of using these strains for biotechnological processes: PHA, H_2_, pigments (PMP), and biomass (OD_660_) (Fig. 7).

When grown in RRNCO medium with CO (1.0 bar) in the absence of light, no variations in hydrogen production among the three strains were observed. The levels of PHB were similar among the three strains: Rr02_01 (11 ± 2 %), 1.7-3A (10 ± 2 %), and 1.4-2B (8 ± 2 %). However, this strain showed a significant increase in the level of PMP (+19%). The maximal OD_660_ reached by strain 1.7-3A was 26% lower than that of the parental strain (0.97 vs 1.30). Concomitantly, the amount of consumed acetate was proportionally lower in strain 1.7-3A, as it consumed one-fourth less of the acetate in comparison to the parental strain (4.1 ± 0.5 mM vs. 5.2 ± 0.8 mM, respectively).

### The genetic variations behind increased CO adaptability in mutants 1.7-3A and 1.4-2B

In order to identify the genetic differences between clones 1.7-3A and 1.4-2B with the parental strain Rr02_01, both mutant genomes were sequenced. A total of 5 and 7 mutations were found in the genome of strains 1.4-2B and 1.7-3A, respectively (Table 1).

**Table 1:** Genetic variations found in strains 1.4-2B and 1.7-3A compared to the parental strain Rr02_01

Strain	Locus/position	CDS	Effect	Nucleotide change	Aa change	Relative posit. (%)*
1.4-2B	Rru_A0933	DegT/DnrJ/EryC1/StrS aminotransferase	FS	G_7_ > G_6_	-	34
	Rru_A1264	Short-chain DH/reductase	FS	C_8_ > C_9_	-	7
	Rru_A1424	NADH DH (ubiquinone) (CooU)	Sub	G > A	A > T	90
	3,782,100	Intergenic Region	-	T_2_ > T_3_	-	-
1.7-3A	Rru_A0625	PpaA (PpsR anti-repressor)	FS	G_6_ > G_7_	-	18
	Rru_A0741	Histidine kinase	Sub	C > T	G > S	76
	Rru_A1265	DeoR family transcriptional regulator	Del	(CGC)_5_ > (CGC)_4_	P_5_ > P_4_	91
	Rru_A1576	Prolyl-tRNA synthetase	Sub	C > T	T > I	49
	Rru_A2602	Diguanylate cyclase/phosphodiesterase	Sub	G > A	G > E	47
	3,820,243	Intergenic region	-	C > A	-	-
Both	Rru_A0697	Multi anti extrusion protein MatE	FS	G_3_CG_1_ > G_3_CG_2_	-	41

*The relative posit. (position) (%) refers to the relative position of the first amino acid affected by the mutation with respect to the N-terminus

*Del*, deletion; *FS*, frame shift; *Sub*, substitution

In strain 1.4-2B, five mutations were found, impacting on a DegT/DnrJ/EryC1/StrS aminotransferase (Rru_A0933), a short-chain dehydrogenase/reductase (Rru_A1264), a NADH dehydrogenase (ubiquinone) (Rru_A1424), and an intergenic region (position 3,782,100). Locus Rru_A1424 (*cooU*) is part of the *cooMKLXUH* operon, which codes for CO-induced hydrogenase-related proteins under the control of the transcription factor CooA (Fox et al. 1996). This mutation does not annul the protein but substitutes an alanine (a hydrophobic amino acid) at position 155 for a threonine (a polar amino acid), with phenotypic consequences that are difficult to predict. Additionally, a mutation affected the gene coding for a member of the subfamily of multidrug and toxic compound extrusion (MATE)-like proteins (Rru_A0697). These proteins are involved in basic mechanisms for homeostasis maintenance by facilitating the excretion of metabolic waste products and xenobiotics (Moriyama et al. 2008).

In the case of strain 1.7-3A, seven differences in its genomic sequence with respect to the parental strain were found. This included a signal transduction histidine kinase (Rru_A0741), a DeoR family transcriptional regulator (Rru_A1265), a periplasmic sensor diguanylate cyclase/phosphodiesterase (Rru_A2602), a prolyl-tRNA synthetase (Rru_A1576), and an intergenic region (position 3,820,243). More interestingly, in this strain, a reversion of a single nucleotide deletion was produced in locus Rru_A0625. This locus codifies a particular version of the anti-repressor PpaA, which in PNSB modifies PpsR repressor activity. PpaA in *R. rubrum* has been recently proved to affect various biological processes related to adaptation to microarobiosis/anaerobiosis (Godoy et al. 2023b). Notably, the same mutation at the Rru_A0697 locus present in stain 1.4-2B was also identified in 1.7-3A. It is worth highlighting that both strains come from completely independent clones.

In general terms, mutations present in 1.4-2B seem to be more related to genes coding for metabolic enzymes, some of them related to redox reactions (dehydrogenases/reductases), whereas mutations present in 1.7-3A are more related to regulatory proteins (anti-repressor, histidine kinase, diguanylate cyclase, etc), showing that the two genetic profiles selected after CO selective pressure are clearly differentiated.

## Discussion

The synthesis gas or syngas is a mixture of mainly CO, H_2_, and CO_2_ that has many applications in the field of biofuels and biomaterials (Drzyzga et al. 2015). From its components, the most challenging to promote cell growth is CO, since it exerts a toxic effect on most (micro)organisms (Robb and Techtmann 2018). For this reason, in this work, we delved into the tolerance and response of *R. rubrum* to this controversial gas. We basically considered two different growth conditions: with and without light. Each scenario showed its own particularities.

### Adaptation to CO might be more limited by metabolic constraints than genetic regulation

As a carbon source, acetate has been shown to promote PHB accumulation and yields better results than other carbon skeletons (such as malate) for this purpose (Narancic et al. 2016). However, acetate may also pose some challenges for *R. rubrum* metabolism. Redox imbalance is mentioned as a possible cause of growth stress in photoheterotrophic growth when acetate is the main carbon source. This stress is more pronounced at high light intensities (150 μmol·m^−2^·s^−1^) and is attenuated when bicarbonate (a source of CO_2_) is available at concentrations around 50 mM (Leroy et al. 2015). Additionally, a functional isoleucine biosynthesis pathway constitutes a key element to mitigate the excess of reducing equivalents (Bayon-Vicente et al. 2020).

When cells are exposed to light, anoxygenic photosynthesis (APS) provides an unlimited energy source, keeping ADP phosphorylation gears in constant movement: the ATP synthases coupled to proton gradient permanently replenish this molecule providing a constant supply of energy (Kim et al. 2017). APS can also be coupled to cofactor recycling by means of reverse electron flow (ERF) (Klamt et al. 2008). If CO is present, it can provide an extra source of energy. By means of the water-gas shift reaction catalyzed by CO dehydrogenase (CODH), electrons from CO are harnessed to promote anabolic reactions. CooF is a membrane-associated iron-sulfur protein tightly associated with CODH. It transfers electrons to a hydrogenase, promoting an exergonic (20.1 kJ·mol^−1^) coupling of CO oxidation with H_2_ production, thus enabling *R. rubrum* to extract energy from CO (Drennan et al. 2001).

However, when cells grown photoheterotrophically (pre-culture) are transferred to a dark environment with CO, the supply of energy is suddenly interrupted. This scenario demands a completely new strategy to keep metabolism rolling. The response to CO is mediated by the transcription factor CooA, which activates CODH, a CO-tolerant hydrogenase, and a collection of genes required to support their correct functioning (Aono 2003; He et al. 1996; Shelver et al. 1995). We have seen a dose-dependent lag phase promoted by CO. On the contrary, even though a slight ppCO-dependent decrease in the growth rate is observed, its variation is not as dramatic as in the case of the lag phase, meaning the toxic effects of CO are mainly observed during the adaptation. However, the genetic response exerted by CooA has been described to act immediately after CO sensing (Roberts et al. 2005). These facts together suggest that the challenge after exposing cells to high levels of this gas arises from difficulties in adjusting some key variables of *R. rubrum* general metabolism, such as redox poise, rather from a toxic effect, especially for cells grown in darkness. This might not be the case for autotrophic cultures in the presence of CO, CO_2_, and H_2_.

Regarding the products with biotechnological interest observed in this study, i.e., PHB and PMP, different conclusions can be stated. As expected, PMP was optimal under light conditions with an initial ppCO of 0.6 bar, which was also optimal for biomass production. Regarding PHB, the titters of this polymer were quite low both in darkness and under light. In relative terms (% of CDW), the PHB was slightly higher in the absence of light, where it was insensitive to the initial amount of CO. These results are consistent with those obtained by Revelles (Revelles et al. 2016). While in phototrophic conditions, PHB mobilization is triggered after the exhaustion of the main carbon source (acetate); in darkness, this polymer can be exploited as a source of energy after CO depletion. Alternatively, PHB mobilization could probably be a consequence of the cease of acetate inward fused by CO exhaustion, as in the case of light. This mobilization was slower in darkness, where the polymer virtually disappeared after 2–3 days of CO depletion, releasing acetate to the medium.

Finally, while in our experimental setup, the amount of CO is controlled only at the beginning of the assay, at a bioreactor scale, the amount of CO can be perfectly controlled. The challenge of adapting *R. rubrum* to high doses of CO may be relevant in the initial steps of a bioprocess. Once adapted, a culture could theoretically be maintained steadily for long periods or even accept higher doses of CO (Rodríguez et al. 2021; Mongili and Fino 2021). However, other limitations observed in our experiments have a greater impact on the design of a bioreactor setup. For example, we have observed that net CO_2_ fixation is virtually non-existent while CO is present, and this fact is independent of how well the cells are adapted to CO. Thus, our results provide useful data as a starting point for designing bioreactor strategies.

### Diauxic curves and growth inhibition at low and high CO levels, respectively, demonstrate that CO can block net CO_2_ fixation

Cultures grown under light presented a shorter lag phase compared to those grown in darkness, and an optimal growth rate at ppCO of 0.6 bar. APS seemed to control global metabolism, speeding adaptation to CO and so, its transformation to CO_2_ and H_2_. Notably, acetate had a fundamental role in this transformation. When it was over, biomass formation virtually ceased. That occurred in cultures with ppCO equal to or higher than 1.0 bar. Small amounts of CO were able to block CO_2_ fixation. Probably, the limiting factor was the uptake hydrogenase (Hup), which has been described as CO-sensitive (Maness et al. 2002). Electrons coming from H_2_ appear to be crucial for net CO_2_ fixation. CO_2_ is a much more oxidized compound (+4) than acetate, making electrons coming from the ERF energetically insufficient to sustain anabolism from this gas. This implies that net CO_2_ fixation differed from CO utilization, stating a great challenge for those biotechnological approaches that pursue fixing carbon from syngas using *R. rubrum* as a biocatalyst. Seeking a heterologous CO-tolerant uptake hydrogenase to replace that from *R. rubrum* could be an interesting way to overcome this limitation.

Phototrophic cultures where CO was completely consumed before acetate depletion (ppCO 0.2 to 0.8 bar) presented two growth phases, with an adaptation lapse in between, describing a typical diauxic behavior. The second growth phase was much slower and finished after the H_2_ was completely consumed. In this case, strain Rr02_01 compared to strain S1 could grow faster and reach a higher OD_660_. The higher PMP at the end of the first exponential phase, which had been the reason why it was preferred over strain S1, was magnified during the second growth phase. Moreover, its OD_660_ did not decrease after reaching its maximum as was the case of S1. This interesting behavior was not expected and suggests that PpaA, which is the fundamental difference between strain Rr02_01 and its parental strain S1, not only serves as anti-repressor for the synthesis of the photosynthetic apparatus, but also maintains its expression level within a defined range, which may not always reach the maximum.

### Strains from the UVa-ALE show *R. rubrum* can be adapted to hostile conditions and some of their mutations suggest a natural ad hoc mutagenesis system

We designed a protocol to isolate clones that adapt more quickly to CO, using UV as mutagen to increase genetic diversity. The strategy was successful, leading to a reduction of the lag phase to the half of strain Rr02_01. The clones selected were sequenced, and the results showed two different escapes to the external pressure exerted by CO on *R. rubrum* physiology. The adaptation of strain 1.4-2B seems to pivot on enzymes with a metabolic function, specially two dehydrogenases, a fact that would contribute to the hypothesis of the general metabolic re-arrangements that must be put into operation to restart growth. Moreover, one dehydrogenase is at the core of CO metabolism: the CO-tolerant dehydrogenase. Rru_A1424 (cooU) is located immediately upstream of cooH, coding for the large subunit of the CO-induced hydrogenase. CooU is a protein of 172 amino acids (18.7 kDa, pI of 5.13) that shows similarity to subunits of the energy-conserving NADH-quinone oxidoreductase complex in various organisms, such as Nqo5 from *Paracoccus denitrificans* (Fox et al. 1996). Although the function of CooU is still unclear, it is described as part of the CO-oxidizing hydrogenase complex, responsible for energy conversion from CO to a proton gradient, ultimately allowing the phosphorylation of ADP (Hedderich and Forzi 2005; Schoelmerich and Müller 2020). As this complex is crucial for CO metabolism, it is unlikely that the A155T substitution leads to a nonfunctional CooU. The mechanisms by which this mutation offers an advantage for growth at high levels of CO are difficult to predict, making it interesting for a devoted study of CooU.

Instead, strain 1.7-3A must have overcome CO pressure after modifying a group of regulatory genes (PpaA-like protein, histidine kinase, and DeoR family transcriptional regulator) and signal transduction systems (diguanylate cyclase/phosphodiesterase), potentially affecting several targets with only a few “shots” (mutations). Both strains presented a shorter lag phase, although other relevant variables were also affected. In the case of strain 1.7-3A, lower levels of OD_660_ were reached compared to the parental Rr02_01, while mutations in strain 1.4-2B impacted positively in PMP.

Delving deeper into the mutations found in both isolates, some striking facts emerged. First, the genome sequence of both UV-mutant strains 1.4-2B and 1.7-3A revealed mutations with notable similarity: some loci had a single nucleotide insertion/deletion (indels) within a sequence of several (7 to 9) consecutive G(C) that shifted the reading frame, resulting in a nonfunctional protein, or even restoring a pre-existing frame-shifted gene (as in the case of PpaA coding sequence). The association between repetitive nucleotides and indels has been previously described: these kinds of sequences are prone to sustain strand slippage mutations (Canceill et al. 1999), which tend to cause small indels (Torres-Cruz and Van der Woude 2003). The regulator PpsR is, in many PSNB, a PpaA partner (Elsen et al. 2005; Kovács et al. 2005). It is responsible for repressing genes related to photosynthesis when oxygen is present. Its activity is controlled by PpaA, which eventually serves as an anti-repressor after sensing a redox signal if oxygen tensions fall under a certain threshold. PpaA can affect PpsR activity, triggering the production of photosynthetic machinery. All these evidences together suggest that in *R. rubrum* genome, repetitive G(C) sequences, which are the common denominator in four mutations, can easily mutate as a measure of last resource when an excessive selective pressure jeopardizes survival. We specifically refer to the three indels in G/C repetitive sequences impacting on loci Rru_A0933 and Rru_A1264 from strain 1.4-2B, Rru_A0625 from strain 1.7-3A, and Rru_A0697 from both strains. This hypothetical mechanism could be responsible for both the emergence of the spontaneous mutant R02_01 by annulling the Rru_A0625 locus (Godoy et al. 2023b) and restoring the same gene after the UVa-ALE protocol. This hints at the possibility that not all mutations present in both strains were induced solely by UV radiation, but may have arisen from other adaptive mechanisms during the mutagenesis experiment. It would be interesting to conduct a more in-depth study to explore whether the adaptability of *R. rubrum* is also rooted in a targeted, *ad hoc* mutagenic mechanism to cope with harsh conditions, such as high levels of CO*.*

In conclusion, we characterized the response of *R. rubrum* to the highest levels of CO ever tested for this bacterium and found a response deeply dependent on the presence or absence of light. The most affected variables were the lag phase in darkness and the growth rate under light. The capacity to transform CO into CO_2_ and H_2_ was limited by the presence of an extra carbon source (mainly acetate but also yeast extract), and the capacity to phototrophically fix CO_2_ into biomass depended on the absence of CO. This dependency delineated a typical diauxic growth curve, which reached higher levels of biomass in the strain Rr02_01 compared to the parental strain. Finally, a UVa-ALE protocol to isolate clones with a shorter lag phase was successfully implemented, although the selected mutations also impacted other relevant variables such as the biomass and the PMP.

## Supplementary information

ESM 1 (PDF 843 kb)

## Data Availability

The datasets generated during and/or analyzed during the current study are available in the accession numbers provided and from the corresponding author on reasonable request.
