# Supplementary material for: Exploring Rhodospirillum rubrum response to high doses of carbon monoxide under light and dark conditions
Source: Appl Microbiol Biotechnol. 2024 Mar 11;108:258. doi: 10.1007/s00253-024-13079-5 (PMC10927898; doi:10.1007/s00253-024-13079-5)
Supplement: Supplementary file 1 [file 253_2024_13079_MOESM1_ESM.pdf]

SUPPLEMENTARY MATERIAL FOR RESEARCH ARTICLE - Applied Microbiology and Biotechnology

Exploring *Rhodospirillum rubrum* response to high doses of carbon monoxide under light and dark conditions.

Manuel S. Godoy [1, 2,*], Irene Verdú[1,‡], Santiago R. de Miguel[1, 2], José D. Jiménez[1, 2], M. Auxiliadora Prieto[1, 2,*]

[1]Polymer Biotechnology Lab, Biological Research Centre Margarita Salas, Spanish National Research Council (CSIC), Madrid, Spain.

[2]Interdisciplinary Platform for Sustainable Plastics towards a Circular Economy-CSIC (SusPlast-CSIC), Madrid, Spain.

[‡]Current address: Drexel University, Philadelphia, Pennsylvania, United States.

*Corresponding authors: auxi@cib.csic.es, msgodoy@cib.csic.es

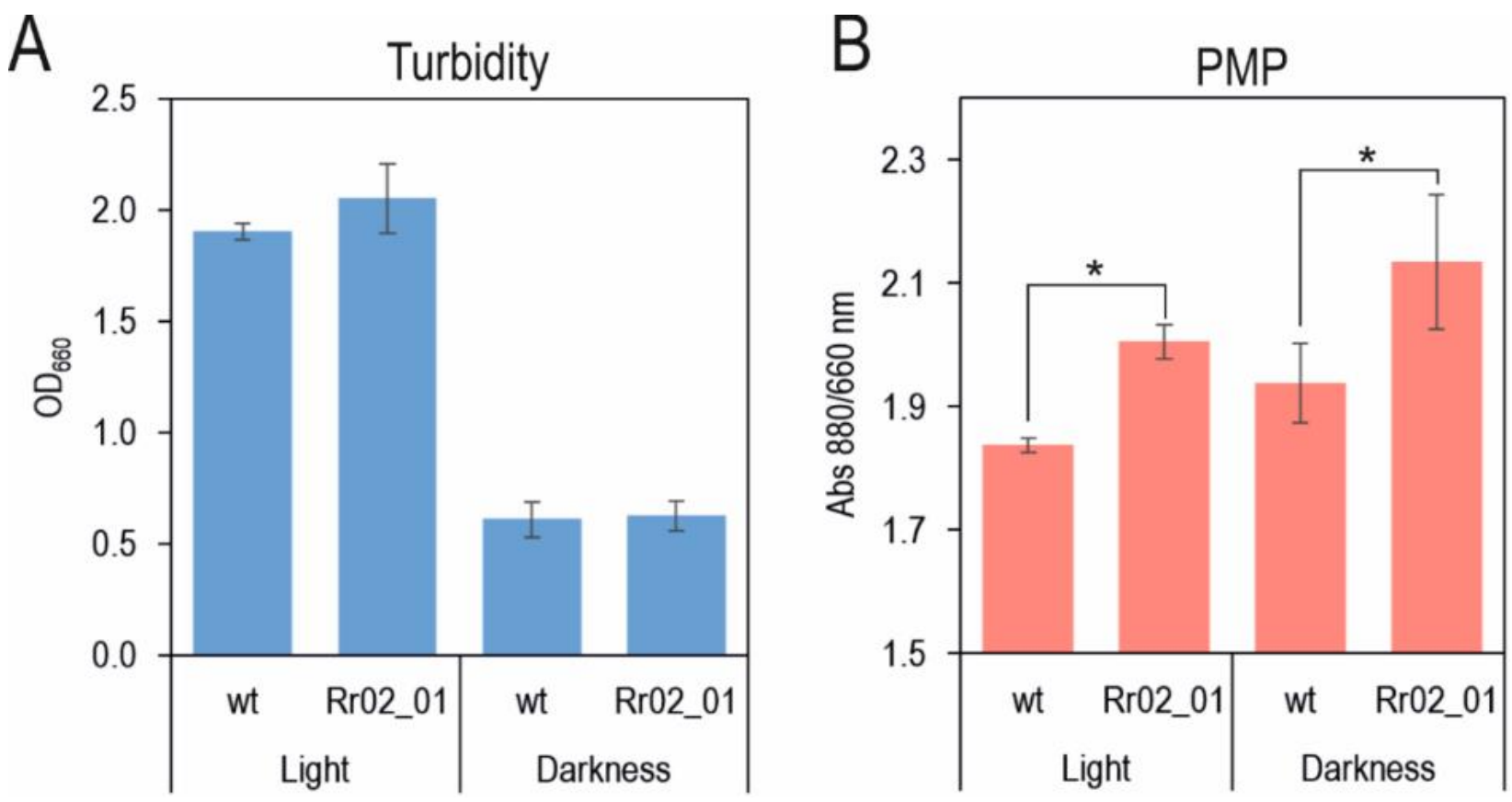

Fig. S1: Comparison between strains Rr02_01 and wild type (S1). Precultures were grown anaerobically in RRNCO medium under light without CO. The inoculum was transferred to the same medium but with ppCO 0.6 bar, and grown under light or darkness. When the acetate was depleted, the turbidity (A) and the PMP (B) were determined. Significant differences were observed in PMP under both light and darkness (+9.1% and +10.1%, respectively). Values represent the mean and the error bars the standard deviation of at least three biological replicates. * denotes significance with p-value <0.05 (unpaired two-tailed Student's test).

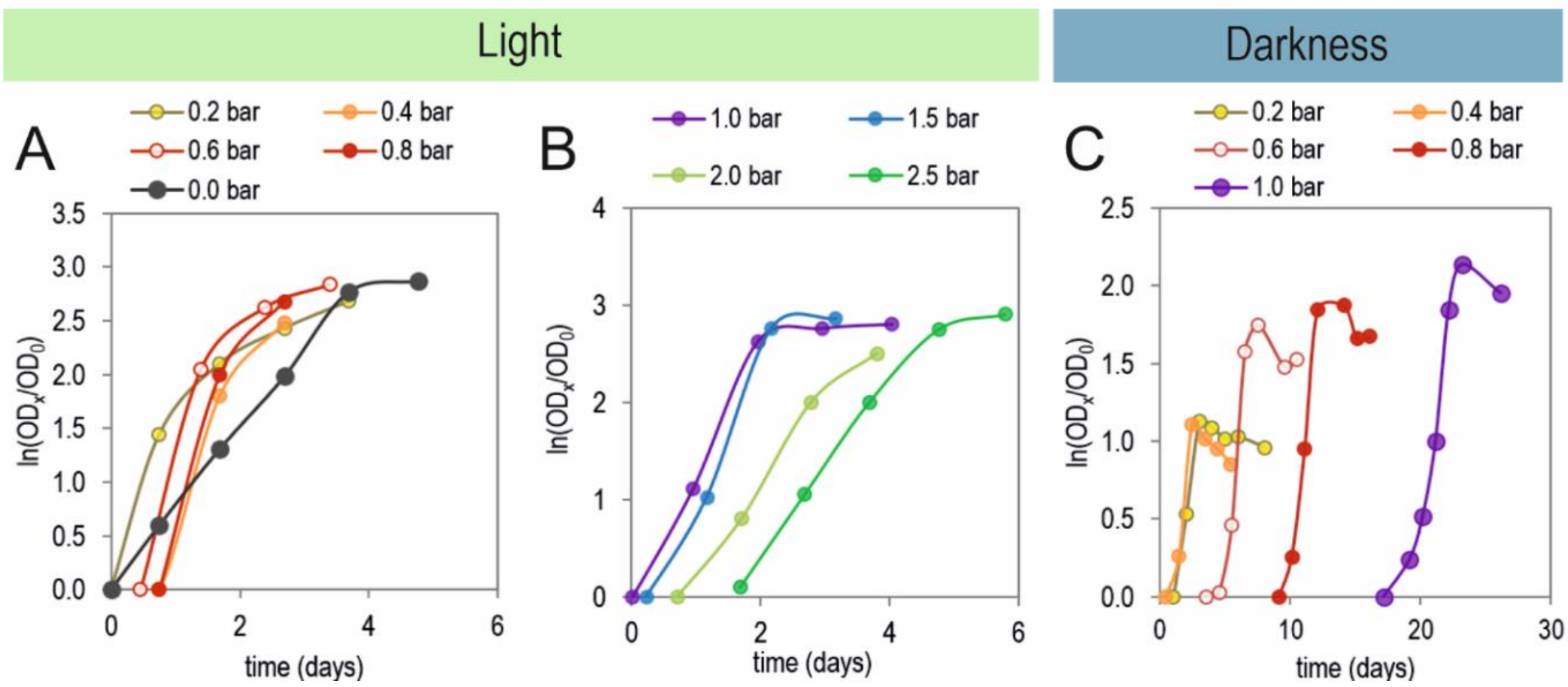

Fig S2. Logarithmic representation of the growth curves under different levels of CO until reaching the stationary phase. Photoheterotrophic cultures grown with an initial ppCO ranging from (A) 0.0 to 0.8 bar and from (B) 1.0 to 2.5 bar. (C) Cultures grown in darkness (initial ppCO ranging from 0.2 to 1.0 bar). Curves represent the $\ln(OD_x/OD_0)$, where OD_x is the OD_{660} at a given time (x) and OD_0 is the initial (x=0) OD_{660}. Standard deviation was less than 10% in all cases (n=3).

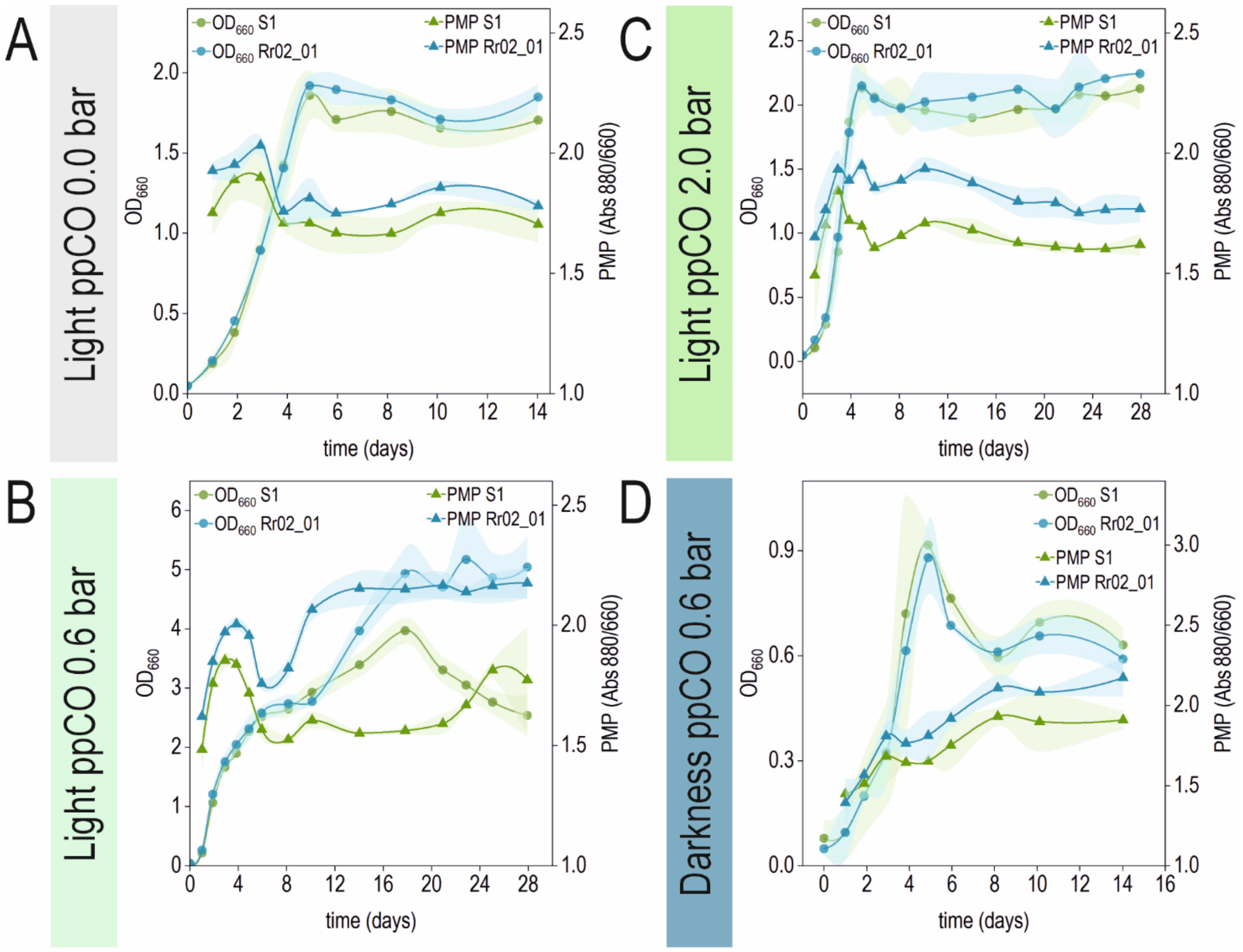

Fig. S3: Comparison between strain Rr02_01 and its parental strain S1. Representative curves of both strains cultured in modified RRNCO medium at different initial ppCO. Photoheterotrophic cultures were conducted with an initial ppCO of 0.0 bar (A), 0.6 bar (B) or 2.0 bar (C). Cultures in darkness were evaluated with ppCO of 0.6 bar (D). As expected, PMP was higher for strain Rr02_01, whereas the turbidity was quite similar for all the conditions. The exception was the evolution of the biomass under light with ppCO 0.6 bar. The second growth phase in strain Rr02_01 reaches much higher levels of OD_{660} and the difference of the PMP is the highest compared to the other three conditions. The shadow denotes the standard deviation (n = 3).

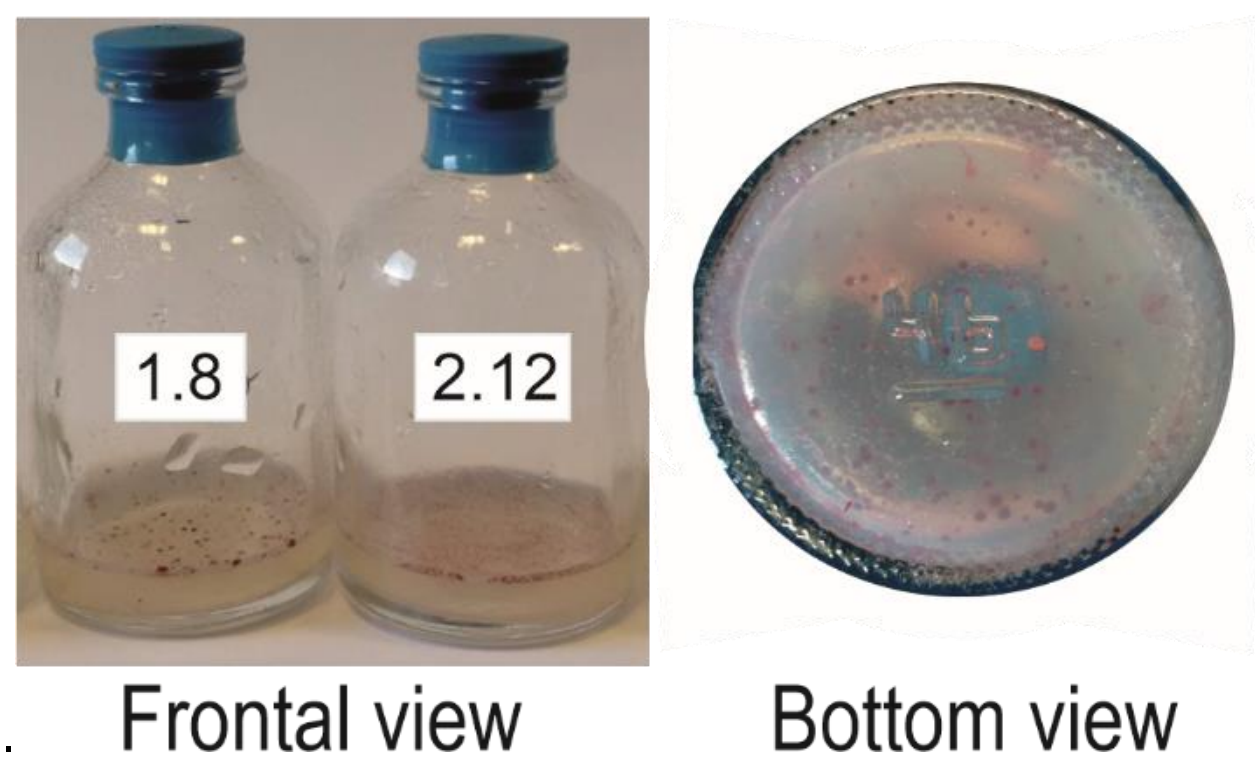

Fig. S4: Pictures of the bottles used to isolate UV-mutant clones. Frontal and bottom views of the bottles containing the solid medium (RRNCO-Acetate 10 mM, agar 1.5% m/v) were filled with CO (0.8 bar) to maintain the selective pressure during this step. Colonies can be observed on the surface of the agar.

Table S1: Type of lamps and intensities used in different labs

Type of lamp	Manufacturer	Product description	Intensity*		Reference
			lux	$\mu mol \cdot m^{-2} \cdot s^{-1}$	
LT	Philips	Master TL-D 18W/865	2300	31	This work
LED	Sencys	10 W, 100 lm, 2650 K	2650-7950	50 - 150	1
LT	Osram	Dulux L 55 W/865, 6500 K	7400	100	2
LT	Osram	Daylight tubes L 15 W/865	1500 - 9000	20-122	3
TB	uns.	uns.	750	15	4,5
TB	uns.	60 W	1000	20	6
HL	uns.	60 W, 750 lumens, soft white	2840	40	7

LT, luminescent tube; TB, tungsten bulb; HL, halogen lamp; uns, unspecified.
*Values colored in blue were calculated using the following equivalences: LT) 74 lux = 1 $\mu mol \cdot m^{-2} \cdot s^{-1}$; TB) 50 lux = 1 $\mu mol \cdot m^{-2} \cdot s^{-1}$; HL) 71 lux = 1 $\mu mol \cdot m^{-2} \cdot s^{-1}$ (Ref. 8), for the sake of comparison. LED coefficient was estimated in 53 using the on-line calculator of Ref. 9.

Table S2: Oligonucleotides used in this study to corroborate mutations in strains 1.4-2B and 1.7-3A

Strain	Position	Locus	Sequence	
			Forward	Reverse
1.4-2B	1,111,548	Rru_A0933	CGAGTTCGCCCGCTATTGT	CCAGGGCTCCCAGATTCTTC
1.4-2B	1,486,030	Rru_A1264	TGTTGACGATCACCCCCTTG	TCACACGTCGGCTCGATAAG
1.4-2B	1,684,048	Rru_A1424	CCATCATGACGCTGTTTCGC	AAGTTTGCGGATGGCTGTTG
1.4-2B	3,782,100	-	CCACCCGATGCCCCATGC	GCTCATCAGCCTTGCTCGC
1.7-3A	742,958	Rru_A0625	GATCGAGAACGAGATTATTCC	GGCCCCCATAGACCGACGC
1.7-3A	866,180	Rru_A0741	GGGAAGGTCAGGGTCCACAG	TACGACATTCAAAGCGAACC
1.7-3A	1,487,040	Rru_A1265	GTTCGCCATCCTGAAATGTCG	CCGATCATTCTCGTTGCC
1.7-3A	1,857,111	Rru_A1576	GCGGAAATCGTCTCCCATC	AGGAATCGACCAGCGGCT
1.7-3A	3,025,390	Rru_A2602	CGCCTGGAAAGCCACATCACC	AGATCGCCAAGCAGAACGAG
1.7-3A	3,820,243	-	GACATAGGCATTTCCATAGA	GGTGGTGGTTATCCGTG
Both	826,816	Rru_A0697	TGGCTGATCCTTTGGCATAC	CCCATCATCATCATCGAGGC

Table S3: Growth parameters of strains S1 and Rr02_01 grown under light with an initial ppCO of 0.6 bar

Parameter	S1	Rr02_01	T-Student
μ_{max} (day^{-1})	1.78 ± 0.03	1.71 ± 0.13	4.19E-01
μ_2 (day^{-1})	0.045 ± 0.004	0.066 ± 0.009	2.61E-02
Final OD_{660}	4.0 ± 0.2	4.9 ± 0.4	2.73E-02
Final PMP	1.56 ± 0.01	2.15 ± 0.06	7.62E-05

μ_2; growth rate of the second growth phase.
The final OD and PMP were calculated at the end of the second growth phase (~18 days).
Numbers in red denote significance (p-value < 0.05, n=3)

References

1. Bayon-Vicente G, Marchand E, Ducrotois J, Dufrasne FE, Hallez R, Wattiez R, Leroy B, (2021). Analysis of the Involvement of the Isoleucine Biosynthesis Pathway in Photoheterotrophic Metabolism of *Rhodospirillum rubrum*. Front Microbiol, 12:731976. https://doi.org/10.3389/fmicb.2021.731976
2. Kaftan D, Bína D, Koblížek M. (2019). Temperature dependence of photosynthetic reaction centre activity in *Rhodospirillum rubrum*. Photosynth Res 142, 181–193. https://doi.org/10.1007/s11120-019-00652-7
3. Porter J, Merrett MJ, (1972). Influence of Light Intensity on Reductive Pentose Phosphate Cycle Activity during Photoheterotrophic Growth of *Rhodospirillum rubrum*. Plant Physiol, 50, (2). https://doi.org/ 252–255. https://doi.org/10.1104/pp.50.2.252
4. Grammel H, Gilles ED, Ghosh R (2003). Microaerophilic cooperation of reductive and oxidative pathways allows maximal photosynthetic membrane biosynthesis in *Rhodospirillum rubrum*. Appl Environ Microbiol. 69(11):6577-86. https://doi.org/10.1128/AEM.69.11.6577-6586.2003
5. Carius AB, Henkel M, Grammel H, (2011). A glutathione redox effect on photosynthetic membrane expression in *Rhodospirillum rubrum*. J Bacteriol, 193(8), 1893-900. https://doi.org/10.1128/JB.01353-10
6. Hosseini SS, Aghbashlo M, Tabatabaei M, Younesi H, Najafpour G, (2015). Exergy analysis of biohydrogen production from various carbon sources via anaerobic photosynthetic bacteria (*Rhodospirillum rubrum*). Energy, 93(1) 730-739. https://doi.org/10.1016/j.energy.2015.09.060
7. McCully AL, Onyeziri MC, LaSarre B, Gliessman JR, McKinlay JB, (2019). Reductive tricarboxylic acid cycle enzymes and reductive amino acid synthesis pathways contribute to electron balance in a *Rhodospirillum rubrum* Calvin-cycle mutant. Microbiol, 166(2), 199–211. https://doi.org/10.1099/mic.0.000877
8. Thimijan RW, Heins RD, (1983). Photometric, Radiometric, and Quantum Light Units of Measure: A Review of Procedures for Interconversion. HortScience, 18(6), 818-822. https://doi.org/10.21273/HORTSCI.18.6.818
9. https://www.waveformlighting.com/horticulture/convert-ppfd-to-lux-online-calculator
